# New Evidence for Retrospectively Cued Perception

**DOI:** 10.3390/vision8010005

**Published:** 2024-02-06

**Authors:** Bence Szaszkó, Moritz Stolte, Lea Bachmann, Ulrich Ansorge

**Affiliations:** 1 Department of Cognition, Emotion, and Methods in Psychology, University of Vienna, 1010 Vienna, Austria; moritz.stolte@univie.ac.at (M.S.); lea.bachmann@univie.ac.at (L.B.); ulrich.ansorge@univie.ac.at (U.A.); 2 Vienna Cognitive Science Hub, University of Vienna, 1090 Vienna, Austria; 3 Research Platform Mediatised Lifeworlds, University of Vienna, 1090 Vienna, Austria

**Keywords:** attentional capture, contrast gain, post-target cueing, retroperception, visual attention

## Abstract

Past research suggests a continuity between perception and memory, as reflected in influences of orienting of spatial attention by cues presented after a visual target offset (post-target cues) on target perception. Conducting two experiments, we tested and confirmed this claim. Our study revealed an elevated reliance on post-target cues for target detection with diminishing target visibility, leading to better performance in validly versus invalidly cued trials, indicative of contrast gain. We demonstrated this post-target cueing impact on target perception without a postcue response prompt, meaning that our results truly reflected a continuity between perception and memory rather than a task-specific impact of having to memorize the target due to a response prompt. While previous studies found an improvement in accuracy through valid compared to invalid cues using liminal targets, in Experiment 1, we further showed an influence of attention on participants’ response time by the post-target cues with cues presented away from a clearly visible target. This suggests that visual interactions at the target location provided no better explanation of post-target cueing effects. Our results generalize prior research with liminal targets and confirm the view of a perception–memory continuum so that visual target processing is not shielded against visuospatial orienting of attention elicited by events following the offset of the visual target.

## 1. Introduction

Whether visual perception arises in a discrete or continuous manner is widely debated. It has long been known that subsequent visual stimuli can alter the perception of a preceding visual target to such an extent that the target cannot be experienced or that it appears drastically distorted in comparison to seeing the target in isolation [1,2,3,4,5,6,7,8]. Such findings suggest a continuity between perception and (short-term) memory [9,10,11,12,13,14,15,16], where the visual perception of a target depends on influences playing out well (i.e., at least up to 400 ms) after target offset so that an initially labile, malleable, and preconscious visual target representation can be altered drastically by influences retrospective relative to the start of target perception and, nonetheless, prior to the conclusion of the target’s perception [17,18]. Critically, this long-lasting perceptual integration window also provides the opportunity for spatial attention elicited by a retrospective or post-target cue (in the following labeled a postcue) to influence the perception of a preceding target even hundreds of milliseconds after target offset [5,6,19].

In general, in retrospective cueing, spatial attention can be directed to one of several possible target locations by a cue that follows the visual target, thus, improving target discrimination performance [5,20,21,22,23,24]. It is relatively undisputed that valid postcueing, presenting a cue at the target position, can improve memory-related target processing, reducing interference during retrieval from iconic memory, short-term memory, or working memory for targets at the cued position [25,26]. For example, studies showing that valid postcueing improved target memory showed no cueing effect on the quality of the stored sensory representation [27,28]. It is, thus, more disputed if target perception is also affected by postcues as would be implied by the continuum of perception and short-term memory.

Here, we, therefore, adopted the promising protocol of Sergent et al. (2013) [5] that seemingly shows postcueing effects on target perception, but took it to the next level and ruled out certain possible complications. To start with a description of the protocol, for their study of the attentional postcueing effect on target perception, the authors used a task in which, in each trial, a single liminal, in their case low-contrast, target was presented at one out of two possible locations. Following the target, the authors presented a non-predictive postcue (a brief dimming of a ring surrounding the target) that was equally likely at the target position (the valid condition; or congruent condition) or opposite of the target (the invalid condition; or incongruent condition). The authors found that the valid postcue improved target discrimination as well as subjective target visibility. This was the case for postcues presented 100 ms, 200 ms, or even 400 ms following the target. Regarding subjective target visibility, Sergent et al. [5] observed that participants judged target visibility to be higher under valid than invalid conditions and that visibility ratings “predicted” (i.e., were positively correlated with) objective target-discrimination accuracy.

Most importantly, Sergent et al. (2013) [5] used liminal targets (i.e., targets with a decreased contrast, near the threshold of target perception), resulting in a retrospective effect on previously presented targets. In this situation, perception of the low-contrast target is delayed [29,30], and, thus, the subjective sequence of target presentation before cue onset can be considerably shorter than intended. For example, by increasing luminance by 1.5 log-units of luminance at threshold, Purushothaman et al. (1998) estimated that perceptual latency was reduced by about 120 ms (from perceiving a stimulus of low luminance and contrast to trail a comparison stimulus by −30 ms to perceiving a stimulus of highest luminance and contrast to lead the comparison stimulus by 80 ms) [29].

Be that as it may, even if the assumption of a sequence of target perception before cue presentation would roughly hold true, using liminal targets only would leave open the important question of if the same type of postcueing influences can be observed with supraliminal targets. Thus, we used supraliminal targets to test if the hypothesis of a perception–memory continuum holds true in general, or if only perception of near-threshold contrast stimuli benefits from post-cueing. If perception is generally continuous with memory, the postcueing effect should be found with supraliminal targets, too.

In addition, Sergent et al. (2013) [5] attributed their effect to the guidance of spatial attention being attracted to the postcues and, hence, to one of the target positions, thus, improving target representations under valid relative to invalid conditions, analogously to pre-cueing [31,32]. However, this conclusion is not entirely certain. The valid cues could have likewise created less perceptual interference than the invalid cues. To understand this, we take a closer look at the procedure of Sergent et al. [5]. As placeholders, these authors used two rings around the possible target positions, and, for the cue, “dimmed” one of the placeholders. In this way, cues could have attracted attention to a previous target position, possibly enhancing whatever perceptual trace of the target was lingering in the observers’ perceptual system. However, as targets were oriented Gabor patches presented inside one of the rings, it is also possible that simply less interfering edge orientation information was present at validly cued than uncued locations at the time of target discrimination. To note, a circular ring provides a mixture of all possible edge orientations, and this information is stronger for a high-contrast ring at an uncued location than for a “dimmed” low-contrast ring at the cued location. Another non-attentional explanation was implied by the study of Xia et al. (2016) [33]. These authors found that cues, as were used by Sergent et al. [5], effectively improved target perception even under conditions of certainty about an unchanging target position, meaning that visual line-orientation interactions between the target and postcues at the target position alone could account for the postcueing effect and that spatial orienting of attention is not decisive. In contrast to a masking account, however, Xia et al. [33] argued for an enhancing effect of postcues on target-orientation perception.

A further caveat concerns the usage of a response prompt in prior demonstrations of postcueing effects on target perception [5,6]. In Sergent et al. (2013) [5], participants were only allowed to judge their target perception well after the target and postcues. This was indicated to the participants by a response prompt. The problem with this procedure is that it is then unclear if the usage of the response prompt corrupted an otherwise perceptual task and changed it into a memory task merely by means of this characteristic of the experimental protocol. Theoretically, if the perception–memory continuum hypothesis holds true, memory should always be involved in perception, not only if the task requires this by means of a post-target response prompt. In the current study, we, therefore, set out to test if the findings of Sergent et al. [5] were also in line with the more general perception–memory continuum hypothesis. To that end, no response prompts were used.

Hence, in the current study, we tested once again whether the postcue can make a difference in target perception and whether postcue-elicited orienting of attention is involved in the effect. To that end, we used cues at one of several possible target positions. Such peripheral cues are among the stimuli that most reliably capture attention [31,32]. Thus, if target perception under invalid conditions suffers from attention being attracted to the cue and, in turn, away from the target, this can be tested by comparison of performance in the invalid condition to that in a neutral condition without a cue. If postcueing effects are indeed based on orienting of attention, as Sergent et al. (2013) [5] assumed, we would expect lower target perception performance under invalid than under neutral conditions as only an invalid cue would direct attention consistently elsewhere.

Most importantly, in Experiment 1, we used clearly visible targets of high contrast (Michelson contrast = 0.94). Only by using such supraliminal visual targets can we test the generality of the perception–memory continuum hypothesis, that is, if memory is also involved in the perception of relatively easily perceived, high-contrast targets. We think that using high-contrast targets is also important to make sure that target perception is not delayed to an extent that could possibly diminish or, in the worst case, even reverse the perceptual order of the objective target–postcue sequence (in which targets were always shown before postcues). Finally, we removed the post-target reporting prompts that Sergent et al. (2013), Thibault et al. (2016), and Xia et al. (2016) used [5,6,33]. These prompts might have artificially converted a perceptual task into a memory task. However, according to the perception–memory continuum hypothesis, any perceptual task should involve memory, opening the door for a postcueing effect on target perception even without a response-prompt imposing this memory demand. This, however, can only be tested by allowing participants to judge the visual target immediately once it is presented. We, therefore, left out the response prompts following the postcues, so that participants could judge the target whenever they wanted, as quickly as possible following the target. This has the additional advantage of allowing us to study the impact of the postcues on reaction times to the target. To note, in prior studies, investigating reaction times as a function of post-target validity was pointless as the response prompt would have equated reaction times in valid and invalid conditions [5,6], i.e., participants would have had to wait until response prompts were shown even if they would have been faster in one of the conditions, for example, the valid condition.

However, we understand that not using a response prompt comes with one disadvantage. Some of the target processing, as well as of the overt reactions to the targets, may precede the postcues. This means that, in the current study, we measured the postcueing effect on target perception under relatively conservative conditions. Yet, even this disadvantage creates helpful data. We think that it is interesting to see how often a response to the targets can be given prior to a postcue as corresponding trials could have also diminished postcueing effects in past studies, but without any understanding of their ratio (relative to trials in which target-related judgments would have definitely been slow enough to allow for postcueing effects on target judgments).

For a comprehensive measure of postcueing on target perception, we further took two measures. To us, it was clear that a larger range of performance levels, from worse to better performance, was desirable for a more exhaustive test of postcueing on target perception. The reason is that attentional effects can be more substantial for more difficult perceptual conditions. For example, although several studies have demonstrated that attention increases the overall neuronal responsiveness at the attended spatial location, regardless of perceptual difficulty (upward shift of the psychometric function [34,35]), other studies suggested that attention enhances the neural sensitivity to a stronger degree under more difficult than under easier conditions, that is, more so for low to intermediate target contrasts than for high target contrasts (leftward shift of the psychometric function; [36,37,38]). As we used high-contrast targets (to counter delayed target perception, see above), in the current study, it was not an option to vary target contrast (as was done, e.g., in the staircase procedure of Thibault et al., 2016 [6]). Initially, in Experiment 1, we, therefore, varied stimulus eccentricity to manipulate visual target perception difficulty and provide a more sensitive test for a postcueing influence. To be precise, the position of targets in the visual field and their distance from the fovea can modulate visual processing and attentional allocation, with stimuli farther from the fovea being more difficult to perceive due to lower spatial resolution and stronger crowding effects [39]. By including eccentricity in our design in Experiment 1, we aimed to provide a more comprehensive test of postcueing. However, even with this procedure, we came nowhere near the threshold of target perception and, thus, took an alternative approach to increase the range of perceptual performance levels further, down to liminal or close to chance-performance levels, in Experiment 2. To that end, we used a “noise-masking protocol” [40,41,42], in which we varied the orientation of the noise mask surrounding an orientation-defined target from very different to very similar to the target orientation and asked participants to decide if a target was present or absent. This methodology also provided the opportunity to increase the sensitivity to capture a potential cueing effect on perceptual precision, compared to the two orientations in Experiment 1.

## 2. Experiment 1

### 2.1. Materials and Methods

#### 2.1.1. Participants

A required minimum sample size of 19 participants was determined to achieve 90% power at a significance level of 0.05 and an effect size of 0.8 (Cohen’s *d*; for a two-sided one-sample *t* test). We chose this effect size because Sergent et al. (2013), in their Experiment 1, found large effects (*d* well over 1) in the interaction between stimulus-onset asynchrony (SOA; between target and postcue) and congruency (here, validity), as well as for the respective main effects [5]. Twenty-two psychology students from the University of Vienna, with normal or corrected-to-normal visual acuity and normal color vision, participated in the experiment in exchange for partial course credit. We tested this large number of participants (compared with the required sample size) for two reasons: First, the experiment was divided into two sessions, meaning we had to cater for a potential drop-out of participants not returning for Session 2. Thus, we wanted to ensure we surpassed the critical number of participants. Second, we also accounted for 10% of participants having to be excluded due to a low rate of correct answers.

Three students only completed the first part of the experiment, resulting in their exclusion. We did not have to exclude any participants due to a low rate of correct answers based on the result of a generalized Extreme Studentized Deviate (ESD) sensitive for multiple outliers (we tested for five outliers), leaving us with 19 participants (nine female, *M_age_* = 23.1 years, *SD_age_* = 3.8 years, ranging from 20 to 33 years) included in the final data analysis.

#### 2.1.2. Apparatus, Stimuli, and Procedure

**Figure 1:**
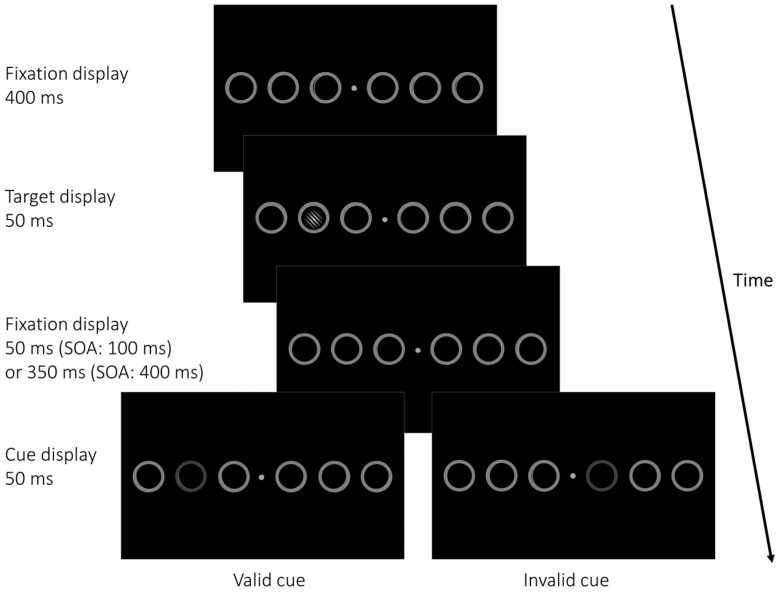
Example of a cued trial in Experiment 1. The six grey placeholder circles remained on screen during an entire block. Each trial started with a fixation display shown for a random duration between 400 and 600 ms before the target (a Gabor patch tilted ±45° from vertical) appeared within one of the six placeholder circles for 50 ms at either 4°, 12°, or 20° eccentricity, measured from screen center. In cued trials, one of the placeholder circles was dimmed, serving as a cue, after a blank (fixation) interval (50 ms/350 ms). Cues could be valid, appearing at the target position, or invalid, appearing at one of three positions on the opposite display side of the target. In neutral (uncued) trials, no cue was presented. Uncued trials served the same function of providing a neutral baseline against which advantages in valid or costs in invalid conditions could be compared, as were the double-cue conditions in Sergent et al. (2013) [5]. Participants had 1 s to indicate the orientation of the Gabor patch, followed by immediate feedback on the screen. SOA = Stimulus Onset Asynchrony. For improved legibility, stimuli are not drawn to scale.

We conducted the experiment in a dimly lit room. Stimuli were presented on a 24.5″ G2590PX AOC Gaming LCD monitor (visible part of the display: 54.4 cm × 30.3 cm), with a resolution of 1920 × 1080 pixels and a refresh rate of 100 Hz. A chin rest ensured a constant viewing distance of 57 cm. The experiment was programmed and executed in OpenSesame 3.3.10 [43], and consisted of a total of 2400 trials, with self-paced breaks after every 120 trials. We divided the experiment into two sessions. Each session started with 60 practice trials. Participants had to report the orientation of a Gabor patch serving as a target (diameter: 2.8°, background-color: L* = 3.3, a* = 0.1, b* = −6.5, Color 1: L* = CIE, L*a*b*, 98.9/1.1/3.3, Color 2: L* = = 3.3, a* = 0.1, b* = −6.5, Michelson contrast = 0.94, linear envelope; see Figure 1), with the buttons “y” (left-tilted by 45°) and “m” (right-tilted by 45°) on a regular ‘qwertz’ keyboard. Instructions stressed both speed and accuracy. Figure 1 shows an exemplary trial, with the relevant stimuli in the target display.

Every trial started with a fixation display with a variable duration between 400 and 600 ms (uniformly distributed), consisting of a black background (CIE L*a*b*, 3.3/0.1/−6.5) and a grey (L* = 54.7, a* = 0, b* = 0) fixation dot with a size of 0.3 × 0.3° in the middle of the screen. From left to right, six grey (L* = 54, a* = 0.5, b* = −0.3) equidistant circles, each with a diameter of 4.2° were positioned, resulting in three circles on each side of the display placed at equal eccentricities (4°, 12°, and 20°) measured from screen center. These circles and the fixation dot remained on the screen for the entire length of each block. Following the fixation display, a target (Gabor patch with a diameter of 2.8° tilted ±45° from vertical) appeared within one of the six placeholder circles for 50 ms at either 4°, 12°, or 20° eccentricity (measured from screen center).

After an SOA of 100 ms or 400 ms (in 50% of cued trials each), a spatial postcue consisting of the dimming of one out of six placeholder circles was shown in cued trials (80% of all trials) for 50 ms, while no cue appeared in the remaining 20% of trials. Postcues were either presented at the target position (valid; 25% of cued trials), or at one of the three positions on the opposite display side of the target position (invalid; 75% of all cued trials, 25% at each possible opposite-display side position). Invalid cues were always shown on the screen side opposite of the target to avoid possibly different effects of centripetal versus centrifugal eye or attention movements as the former are usually faster than the latter [44,45]. Participants had 1 s (measured from target onset) to indicate the target orientation; during that time, and after target offset, the fixation display was shown again (note that the fixation cross and the placeholder circles were always present during the entire experiment, except during breaks); following this interval, and before the next trial began, they received written feedback for 1 s: “Correct!”, “Wrong!”, or “Too slow!”. Trials with and without cues, valid, neutral, and invalid conditions, cue positions, and target positions were presented in a pseudo-randomized sequence with each block.

### 2.2. Results

#### 2.2.1. Accuracy Rates

**Figure 2:**
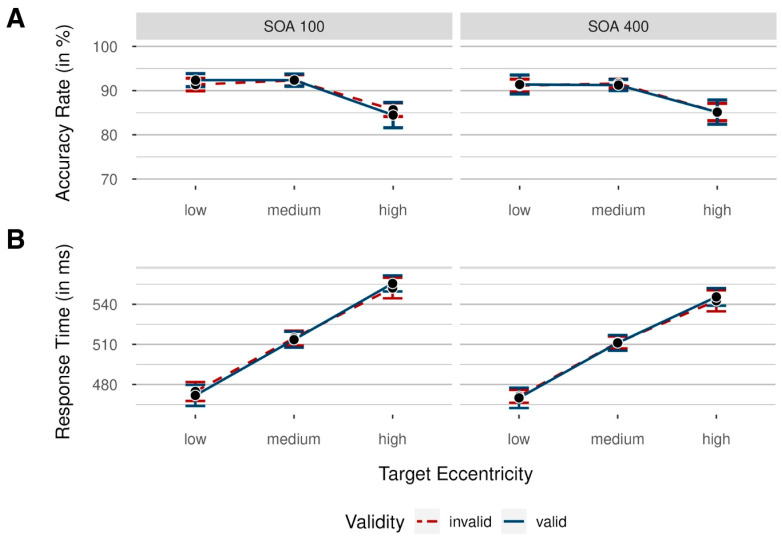
Means of participants’ accuracy rates (**A**) and response times (**B**) in Experiment 1 as a function of target eccentricity (low = nearest to screen center/medium/high = farthest from screen center), colored by validity (invalid = cue shown at a different position than the target: red dashed line/valid = cue and target shown at the same position: blue solid line), for short (100 ms) and long (400 ms) stimulus–onset asynchronies (SOAs) between target and cue. Data were collapsed across both sides of the target display. Error bars indicate 95% CI based on mean values per participant and condition, correcting for within-subject designs.

Accuracy rates were analyzed by conducting a 2 × 3 × 2 repeated-measures analysis of variance (ANOVA), with independent variables validity (valid/invalid; uncued trials were excluded from this analysis), target eccentricity (low/medium/high), and SOA (100 ms/400 ms). In Figure 2, mean accuracy rates (ARs) are shown for each condition and participant.

We found a significant main effect of target eccentricity, *F*(2, 36) = 31.55, *p* < 0.001, $\eta_{p}^{2}$ = 0.64, BF_incl_ > 100. This resulted from significantly worse performance in trials with high target eccentricity compared to trials with medium target eccentricity, with differences tested using a two-sided *t* test versus zero, *M* = −6.78%, *SD* = 4.58%, 95% CI [−8.98, −4.57], *t*(18) = −6.45, *p* < 0.001, $d_{unb}$ = −1.42, BF_10_ > 100. Differences between medium and low target eccentricity were not significant, *M* = 0.32%, *SD* = 2.39%, 95% CI [−0.83, 0.15], *t*(18) = 0.59, *p* > 0.999, $d_{unb}$ = 0.13, BF_10_ = 0.28. Interestingly, all other main effects, as well as interactions, were not significant (all *p*s > 0.290, all BF_incl_ < 0.32), with no significant main effect of validity and no significant interaction between validity and SOA.

Furthermore, we were interested in examining the results for the lowest eccentricities, as this analysis provides the most direct comparison to the results obtained by Sergent et al. (2013) [5], but found no significant main effects or interactions, all *p*s > 0.435, all BF_incl_ < 0.47. In addition, we compared performance in invalidly cued versus uncued trials (valid trials were excluded) to check for effects of attention on target perception with a 2 × 3 repeated-measures ANOVA, with independent variables validity (invalid/uncued) and target eccentricity (low/medium/high). However, neither the main effect of validity nor the interaction between validity and target eccentricity were significant (all *p*s > 0.629, all BF_incl_ < 0.26).

#### 2.2.2. Response Times

Prior to RT analyses, all response times below 150 ms and above 1000 ms were deleted (1.07% of all data). We analyzed response times with a 2 × 3 × 2 repeated-measures ANOVA, again with independent variables validity (valid/invalid), target eccentricity (low/medium/high), and SOA (100 ms/400 ms). Mean response times (RTs) are shown in Figure 2 for each condition.

We found a significant main effect of SOA, *F*(1, 18) = 8.42, *p* = 0.009, $\eta_{p}^{2}$ = 0.32, BF_incl_ = 4.70, mainly driven by faster responses in the long SOA condition, *M* = 509 ms, *SD* = 46 ms, 95% CI [500, 517], compared to the short SOA condition, *M* = 514 ms, *SD* = 50 ms, 95% CI [504, 523]. Analogously to the results above, we found a significant main effect of target eccentricity, *F*(2, 36) = 202.80, *p* < 0.001, $\eta_{p}^{2}$ = 0.92, BF_incl_ > 100, reflecting a performance decrease from low to high target eccentricities. RT differences between low and medium target eccentricities were significant, *M* = 40 ms, *SD* = 14 ms, 95% CI [34, 47], *t*(18) = 12.67, *p* < 0.001, $d_{unb}$ = 2.78, as were differences between medium and high target eccentricities, *M* = 35 ms, *SD* = 15 ms, 95% CI [28, 42], *t*(18) = 10.55, *p* < 0.001, $d_{unb}$ = 2.32.

Interestingly, the main effect of validity was not significant, *F*(1, 18) = 0.01, *p* = 0.937, $\eta_{p}^{2}$ < 0.01, BF_incl_ = 0.28, indicating a general lack of performance differences between validly and invalidly cued trials. Next, we examined results for the lowest eccentricities in isolation, as this analysis again provides the most straightforward comparison to the results obtained by Sergent et al. (2013) [5], but found no significant main effects or interactions, all *p*s > 0.322, all BF_incl_ < 0.61, with the lacking validity effect confirming the conclusions of the main analysis.

Again, we compared performance in invalidly cued versus uncued trials (valid trials were excluded) with a 2 × 3 repeated-measures ANOVA, including the independent variable’s validity (invalid/uncued) and target eccentricity (low/medium/high). Interference through capture by invalid cues was significant, *F*(1, 18) = 13.20, *p* = 0.002, $\eta_{p}^{2}$ = 0.42, BF_incl_ = 6.30, reflected in significantly slower responses in invalidly cued trials, *M* = 511 ms, *SD* = 40 ms, 95% CI [501, 523], than in uncued trials, *M* = 506 ms, *SD* = 40 ms, 95% CI [495, 517]. We found no significant interaction between validity and target eccentricity, *F*(2, 36) = 0.22, *p* = 0.802, $\eta_{p}^{2}$ = 0.01, BF_incl_ = 0.17.

### 2.3. Discussion

In Experiment 1, we did not replicate the cueing effect found by Sergent et al. (2013) [5]: validly cueing the target did not benefit performance relative to invalidly cueing the target. Importantly, however, we found interference by invalid cues relative to neutral cueing conditions without a cue. This was found in RTs but not found in ARs, whereas Sergent et al. observed all of their cueing effects in ARs and in fact were not able to analyze the RTs (as all responses were given after a response prompt) [5]. This finding is remarkable, given that we did not use a postcueing response prompt and, thus, participants were free to judge the target even prior to cue onset. A weaker postcueing effect was therefore to be expected in the current study in the first place as some of the postcues probably occurred too late to influence judgments about target perception. The fact that invalid postcues nonetheless interfered with target responses is, thus, a confirmation of Sergent et al.’s [5] interpretation that post-target cues can modulate target perception through the capture of attention even after target offset. That the corresponding effects were found in RTs rather than in accuracies is probably due to the fact that we used targets well above their visibility threshold. With targets well above the threshold, the question is not so much if the target can be correctly perceived but how quickly this is possible.

For the missing cueing effect between invalid and valid cues, at least two conceivable explanations should be discussed in more detail. First, it is possible that, even though Sergent et al. (2013) adopted dimming cues to minimize the effect of backward masking [5], masking might have been only weaker but not entirely absent in the present experiment, thus, still counteracting beneficial capture effects on target perception to some extent. This would explain why we found interference by invalid cues and not facilitation by valid cues. Second, at least some of the advantages from valid cues in Sergent et al. [5] could have been due to less perceptual interference by dimming rings at target positions rather than to attention capture. This explanation is not unlikely as we did not use the same type of targets close to the threshold, and, thus, undermined those cueing effects due to delayed perception of low-contrast targets in Sergent et al. (2013) [5]. To note, we used high-contrast targets. A third possible explanation is that the dimming of the placeholder circles in the present experiment was not salient enough or was too weak. However, this explanation was clearly refuted by comparing response times between invalid and uncued trials. We found that invalid cues significantly slowed down participants’ responses, indicating not only attentional capture by the cues but also that this kind of capture modulated the perception of the temporally preceding targets. A fourth possible explanation was also unlikely: by presenting cues in only 25% of the trials at the target position, but in 75% of the trials at a display location opposite to the side of the cue, we could have inadvertently invited participants to strategically shift their attention to the side opposite of the cue. However, net disadvantages in invalidly cued compared to uncued trials make this explanation unlikely as it would have been pointless for the participants to strategically shift their attention if this was associated with a processing cost.

Our final potential explanation for the absence of a cueing benefit under valid conditions compared to Sergent et al. (2013) [5] is ceiling performance. Our study used targets well above the visibility threshold, leading to high baseline performance, which may have masked any cueing benefits due to limited room for improvement. In contrast, Sergent et al. observed lower baseline performance, allowing for more improvement [5]. However, we have to mention that only two subjects scored 100% correct in a single condition of the current experiment, limiting the likelihood of this explanation. Nevertheless, Experiment 2 aimed to shed light on this by using different noise orientations to manipulate performance levels, while keeping performance levels in a dynamic range. Specifically, it could be that the postcueing facilitation by valid post-target cues reflects contrast gain [37], and is, thus, stronger for more liminal than well-visible targets. To note, this was the major difference between the present Experiment 1 and Sergent et al. [5]. In Experiment 2, we used a broad range of target visibilities, allowing us to investigate if the postcueing facilitation by valid post-target cues was indeed stronger for or even restricted to targets of lower visibility, a question that was hitherto neither tested by Sergent et al. [5], as they used only targets close to threshold, nor in the present Experiment 1 that used only clearly visible targets well above threshold.

## 3. Experiment 2

### 3.1. Materials and Methods

#### 3.1.1. Participants

Twenty-five psychology students from the University of Vienna participated in the experiment. After excluding one participant due to a low rate of correct answers, again tested using a generalized ESD (testing for four outliers), data from 24 participants (15 female, *M_age_* = 23.0 years, *SD_age_* = 3.0 years, ranging from 19 to 32 years) with normal or corrected-to-normal vision were analyzed.

#### 3.1.2. Apparatus, Stimuli, and Procedure

**Figure 3:**
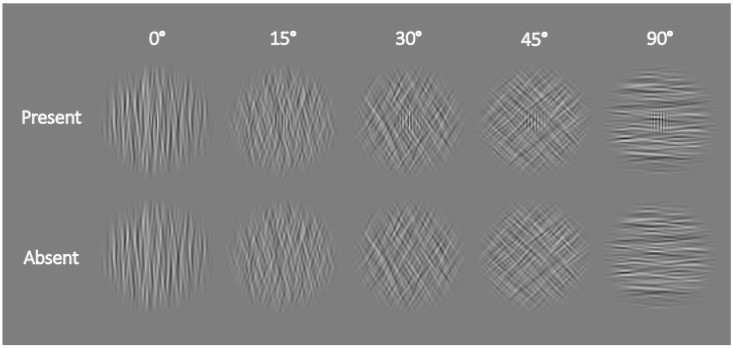
Example stimuli used in Experiment 2. In the top line, stimuli with increasing noise orientation (and decreasing difficulty) from left to right with a target Gabor patch present are depicted; in the bottom line, stimuli with the same noise orientation, but with the patch absent, are shown. From left to right, the stimuli show different degrees of rotation of the noise mask. The Gabor-patch target had a fixed orientation of 0°.

To provide a more comprehensive measure of the postcueing effect, we decided to vary the visibility of our stimuli by embedding vertically oriented Gabor patches as targets in a round noise mask (10% root-mean-square contrast, 2.8° in diameter; for a successful application of such a protocol, see also [42]) varying in orientation, which was band-pass filtered in the orientation domain (10° bandwidth)—see Figure 3 for an illustration. The target was only present in half of the trials and absent in the other half.

Participants had to indicate whether the Gabor Patch (0.9° in diameter) was present or absent within the noise mask by pressing the “up arrow” for present and “down arrow” for absent. The greater the difference between the orientation of the noise mask and the Gabor patch (which was always vertical, thereby having an orientation of 0°), the easier the task. We used five different noise orientations: 0°, 15°, 30°, 45°, and 90°. The positions and sizes of the placeholder circles and the stimuli were the same as those with the nearest eccentricities in Experiment 1. However, we changed the background of the stimulus display to grey (CIE L*a*b*, 54.7/0/0) with a black (CIE L*a*b*, 3.3/0.1/−6.5) fixation dot and black placeholder circles. Analogously, the colors of the Gabor patch were also changed from black and white to black and grey.

**Figure 4:**
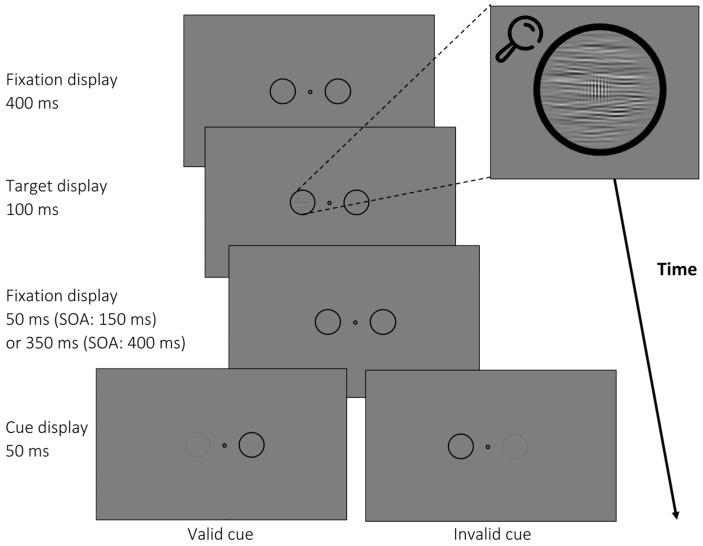
Trial example from Experiment 2. After a variable fixation period, a noise mask with varying orientation (here: 90°) appeared before the cue (dimming of placeholder circle by 50% luminance). A target was presented in the noise mask in 50% of all trials (depicted), but the target was missing in the other 50% of all trials (not depicted). Participants had to indicate whether a Gabor patch was present or absent within the noise stimulus. All placeholders, cues, and targets were presented at 4° eccentricity.

In this experiment, participants completed 1600 trials divided into 10 blocks in a single session. An example of a trial is shown in Figure 4. Target–postcue SOAs were 150 ms and 400 ms. As the target was shown for 100 ms (50 ms longer than in Experiment 1), we had to change the shorter SOA to still maintain an adequate target–cue interval of at least 50 ms. In Experiment 2, 50% of the cues were valid and 50% invalid. In addition, we again used uncued trials (20% of all trials) to compare performance with invalid trials. In contrast to Experiment 1, participants were not required to discriminate target orientations. Instead, they had 1 s to indicate the presence or absence of the Gabor patch by keypress. Again, they received written feedback analogously to Experiment 1.

#### 3.1.3. Data Analysis

In addition to the analyses reported in Experiment 1, we fitted two different psychometric functions to the data using a Maximum Likelihood Criterion because we assumed a possible nonlinear relation between stimulus visibility and accuracy: a Cumulative Gaussian function and a Logistic function. For this, we used mean accuracy rates for each SOA and orientation condition. To this end, we used MATLAB [46] with the Palamedes Toolbox [47]. Note that we could not fit individual models to the data (as individual participants tended to act as noise for the model in question, and, for many of them, corresponding models could not be fitted based on one mean accuracy level per condition alone). In other words, individual participants behaved differently, with some participants contributing and others not contributing to the evidence observed in the average function of psychophysical performance. This is important, as average performance should not be confused with individual behavior, i.e., what is true of the average across humans is, thus, not necessarily what can be observed at an individual performance level.

We report the mean of the parameter estimate of the cut-off of the function on the *y* axis (α), the slope of the function (corresponding to the *SD*; β), the threshold (lower asymptote of the function; γ), and the lapse rate (upper asymptote of the function; λ). We additionally calculated *d*’ per participant and condition by first calculating the hit rate (Equation (1)) as well as the false alarm rate (Equation (2)) and adjusting both using a loglinear correction to avoid extreme values. For the calculation of hit rates (*HR*), we divided the corrected amount of hits (*N_Hits_*) by the corrected sum of hits (*N_Hits_*), misses (*N_Miss_*), and timeouts in trials where a target stimulus was present (*N_TP_*). For the calculation of false alarm rates (*FR*), we divided the corrected number of false alarms (*N_FA_*) by the sum of false alarms (*N_FA_*), correct rejections (*N_CR_*), and timeouts when a target was absent (*N_TA_*). We then *z*-transformed the HRs and FRs and finally subtracted the *z*-transformed FRs from the *z*-transformed HRs.

(1) $$HR\;=\;\frac{\left(N_{Hit}\;+\;0.5\right)}{\left(N_{Hit}\;+\;N_{Miss\;}+\;N_{TP\;}+\;1\right)}$$

(2) $$FAR\;=\;\frac{\left(N_{FA}\;+\;0.5\right)}{\left(N_{FA}\;+\;N_{CR\;}+\;N_{TA}+1\right)}$$

### 3.2. Results

#### 3.2.1. Accuracy Rates

**Figure 5:**
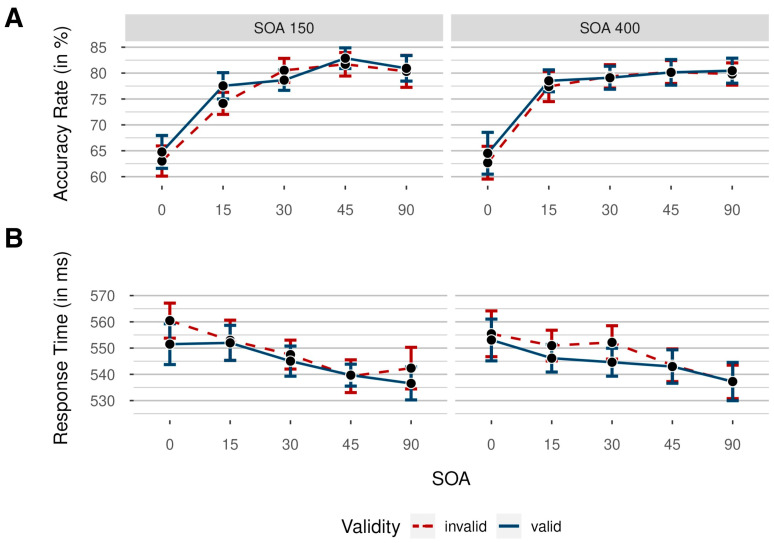
Means of participants’ accuracy rates (ARs; (**A**)) and response times (**B**) in Experiment 2 as a function of noise orientation (from 0° to 90°), colored by validity (red dashed line: invalid/blue solid line: valid), for short (150 ms) and long (400 ms) stimulus–onset asynchronies (SOAs) between target and cue. Colored data points indicate mean AR in the respective condition. Data were collapsed across both sides of the target display. Error bars indicate 95% CI based on mean values per participant and condition, correcting for within-subject designs.

Accuracy rates were analyzed by conducting a 5 × 2 × 2 repeated-measures ANOVA, with the independent variables noise orientation (0°/15°/30°/45°/90°), SOA (150 ms/400 ms), and validity (valid/invalid). Mean accuracy rates for every condition are depicted in Figure 5. The results showed a significant main effect for orientation, indicating decreased difficulty by increased orientation, as intended, *F*(4, 92) = 58.80, *p* < 0.001, $\eta_{p}^{2}$ = 0.72, BF_incl_ > 100. Critically, there was neither a significant main effect of validity, *F*(1, 23) = 2.79, *p* = 0.108, $\eta_{p}^{2}$ = 0.11, BF_incl_ = 0.35, nor of SOA, *F*(1, 23) = 0.27, *p* = 0.607, $\eta_{p}^{2}$ = 0.01, BF_incl_ = 0.17.

We observed a significant interaction between SOA and orientation (although our Bayes Factors indicated otherwise), *F*(4, 92) = 2.66, *p* = 0.037, $\eta_{p}^{2}$ = 0.10, BF_incl_ = 0.46. However, after correcting for multiple comparisons, none of the differences between the respective SOAs (calculated separately for each orientation level) were significant, all *p*s > 0.101. Bayesian *t* tests indicated a difference in accuracy between long and short SOA at 15°, BF_10_ = 2.71, while being ambiguous at 45°, BF_10_ = 1.13. There seemed to be no difference at the other orientations (all BF_10_ < 0.26).

The interactions between SOA and validity, *F*(1, 23) = 0.13, *p* = 0.719, $\eta_{p}^{2}$ = 0.01, BF_incl_ = 0.21, as well as between validity and orientation, *F*(4, 92) = 1.58, *p* = 0.185, $\eta_{p}^{2}$ = 0.06, BF_incl_ = 0.18, and the three-way interaction between SOA, validity, and orientation, *F*(4, 92) = 0.47, *p* = 0.759, $\eta_{p}^{2}$ = 0.02, BF_incl_ = 0.08, were not significant.

An additional repeated-measures ANOVA comparing performance in invalid versus uncued trials, with the independent variables noise orientation (0°/15°/30°/45°/90°) and validity (invalid/uncued), showed neither a significant main effect of validity, *F*(1, 23) = 0.22, *p* = 0.643, $\eta_{p}^{2}$ = 0.01, BF_incl_ = 0.19, nor a significant interaction between validity and orientation, *F*(4, 92) = 1.22, *p* = 0.307, $\eta_{p}^{2}$ = 0.05, BF_incl_ = 0.20. The main effect of orientation was again significant, indicating increasing accuracy rates with increasing noise orientation (and, thereby, decreasing task difficulty), *F*(4, 92) = 55.29, *p* < 0.001, $\eta_{p}^{2}$ = 0.71, BF_incl_ > 100.

**Figure A1:**
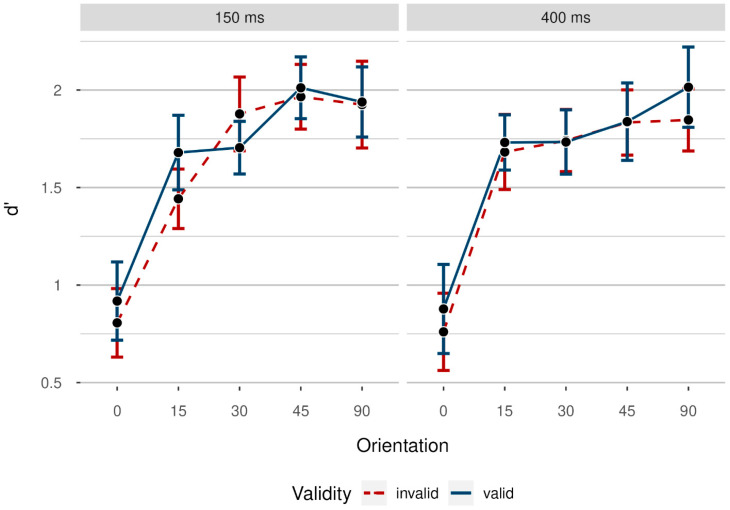
Mean of participants’ *d*’ grouped by noise orientation (from 0° to 90°), colored by validity (red dashed line: invalid/blue solid line: valid), for short (150 ms) and long (400 ms) stimulus–onset asynchronies between target and cue. Colored data points indicate mean *d*’ per participant in the respective condition. Data were collapsed from both sides of the target display. Error bars indicate 95% CI based on mean values per participant and condition, calculated using Morey’s (2008) correction for within-subject designs.

To summarize, we did not observe a significant benefit of post-target cues on accuracy rates: There was no postcueing effect, as well as no significant variation of postcueing effects with increasing task difficulty. There was also no difference between performance in invalid and uncued trials. The results we obtained using *d*’ as a dependent variable painted the same picture (see Appendix A, Figure A1 as well as Table A1 for the results of the analysis).

**Figure 6:**
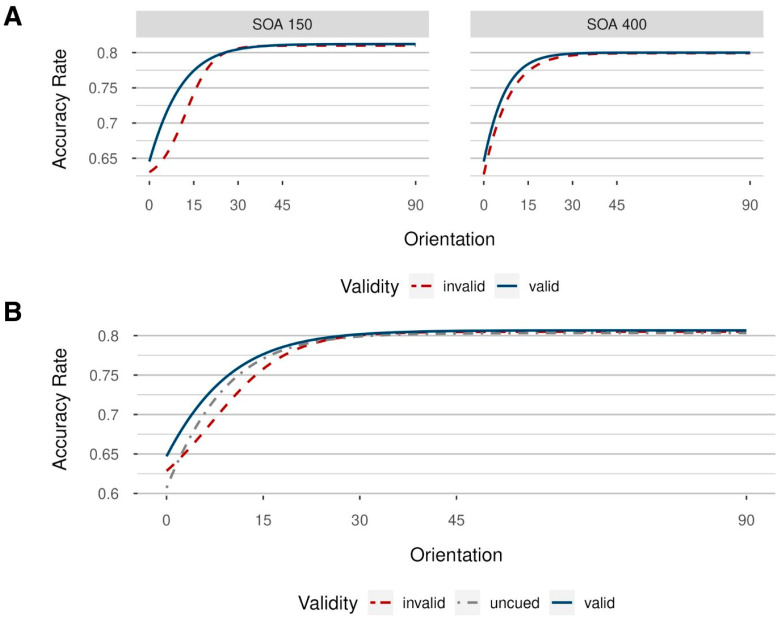
Logistic models fitted to mean accuracy rates across participants in Experiment 2, with varying stimulus orientation (from 0° to 90°); (**A**): one model for each validity condition (blue solid line: valid condition; red dashed line: invalid condition) and stimulus–onset asynchrony (SOA; short SOA = 150 ms on the left, long SOA = 400 ms on the right side). Even in the 150 ms SOA condition, there were no benefits in valid conditions beyond 15° of visual angle, while there were no benefits in the valid trials in the 400 ms SOA condition at all. (**B**): uncued condition (grey dot-dashed line) additionally included, aggregated over stimulus–onset asynchronies (SOAs).

For a second, partly independent, test, we fitted psychometric functions based on the averages across participants, separately for the different combinations of the steps of the independent variables, depicted in Figure 6. Here, the goodness of fit was evaluated using both the AIC and BIC values. Logistic models yielded a better fit to the data than cumulative Gaussian models, as reported in Table A2, Appendix A. Model parameters are reported in Table A3, Appendix A. In both SOA conditions, a leftward shift is visible in valid compared to invalid conditions. Across participants, performance seems to depend on the presence of valid postcues in a manner inversely proportional to the visibility of Gabor patches (with higher performance in valid compared to invalid trials when the visibility of targets is comparatively low), a phenomenon indicative of contrast gain [37]. Importantly, however, one should be careful not to overinterpret Figure 6, as there were probably no validity effects beyond 15° of visual angle, even in the 150 ms SOA condition, while there were no effects in the 400 ms SOA condition at all.

Additionally, we also included uncued trials in our analysis by first collapsing the data over the respective SOAs (as uncued trials had no SOA, contrary to valid and invalid trials) and then fitting a model per validity condition. We expected costs in invalid to uncued conditions, as well as benefits in valid compared to uncued conditions at low-to-medium orientation levels. This is exactly what we found (Figure 6B). Corresponding model parameters are reported in Table A4, Appendix A.

#### 3.2.2. Response Times

Prior to RT analyses, all response times below 150 ms and above 1000 ms were deleted (2.40% of all data). We analyzed response times by again conducting a 5 × 2 × 2 repeated-measures ANOVA, with the independent variable’s noise orientation (0°/15°/30°/45°/90°), SOA (150 ms/400 ms), and validity (valid/invalid). Mean RTs per condition are shown in Figure 5. Importantly, we found a significant main effect of validity, *F*(1, 23) = 9.20, *p* = 0.006, $\eta_{p}^{2}$ = 0.29, BF_incl_ = 0.89. Participants were faster in the validly cued trials, *M* = 545 ms, *SD* = 21 ms, 95% CI [542, 547], than in the invalidly cued trials, *M* = 553 ms, *SD* = 17 ms, 95% CI [545, 551]. As the results of the frequentist and the Bayesian analyses clearly seemed to differ (the first being highly significant, although with negligible effect size, the latter somewhat ambiguous), we followed up with Bayesian and frequentist post-hoc comparisons, both yielding evidence in favor of a significant difference between valid and invalid trials, *M* = 3 ms, 95% CI [1, 5], *SD* = 5 ms, *t*(23) = 2.90, *p* = 0.008, $d_{unb}$ = 0.06, BF_10_ = 1.92.

The main effect of orientation was also significant, *F*(4, 92) = 10.30, *p* < 0.001, $\eta_{p}^{2}$ = 0.31, BF_incl_ > 100, with mean RTs decreasing with increasing noise orientation (and, analogously, with decreasing task difficulty), as expected. The main effect of SOA was not significant, *F*(1, 23) = 0.09, *p* = 0.762, $\eta_{p}^{2}$ < 0.01, BF_incl_ = 0.17. All interactions were not significant, all *p*s > 0.359, and all BF_incl_ < 0.23.

We conducted an additional repeated-measures ANOVA comparing performance in invalidly cued trials versus uncued trials (valid trials were excluded), with two within-participant variables: validity (invalid/uncued) and noise orientation (0°/15°/30°/45°/90°). The main effect of validity failed to reach significance, *F*(1, 23) = 3.52, *p* = 0.074, $\eta_{p}^{2}$ = 0.13, BF_incl_ = 0.58, albeit showing the same trend as in Experiment 1, with higher RTs in invalid trials, *M* = 548 ms, *SD* = 21 ms, 95% CI [544, 552], than in uncued trials, *M* = 544 ms, *SD* = 24 ms, 95% CI [540, 548].

We found a significant main effect of orientation, *F*(4, 92) = 9.92, *p* < 0.001, $\eta_{p}^{2}$ = 0.30, BF_incl_ > 100, indicating faster responses with decreasing task difficulty (higher orientation of the noise), but no significant interaction between validity and orientation, meaning that RT differences between uncued and invalid trials did not vary with increasing task difficulty, *F*(4, 92) = 1.12, *p* = 0.351, $\eta_{p}^{2}$ = 0.05, BF_incl_ = 0.17.

In summary, our results showed significantly faster responses in validly postcued trials than in invalidly postcued trials, indicating a postcueing effect. Although only marginally significant, the trend for slower responses for invalid compared to uncued trials also speaks for postcueing. There was also a significant main effect of orientation, indicative of differences in difficulty between the respective stimulus orientations. However, validity effects did not differ depending on task difficulty.

### 3.3. Discussion

In Experiment 2, we observed improved orientation discrimination performance in valid compared to invalid trials, replicating previous findings on the influence of postcueing on target perception [5,33]. Using a linear model, our effects were only significant for response times, but not for accuracy rates and *d*’. However, when fitting logistic models with orientation as an independent variable, and participants’ mean accuracy rates for both SOA conditions, we found that postcues specifically enhanced behavioral sensitivity for more difficult conditions (lower noise orientations), indicated by a leftward shift of the psychometric orientation discrimination function in valid compared to invalid trials. This was found for relatively extreme masking conditions (below about 15° of the angle of the noise) and in the short SOA (with 150 ms) condition only. This is perfectly in line with a relatively short-lived contrast gain modulation, which is thought to increase the neuronal sensitivity for sensory information depending on perceptual difficulty or stimulus contrast (here: noise orientations) [37,48], but not for extended times past the visual stimulus (i.e., not beyond maybe 150 ms or so). Critically, postcueing effects were accompanied by neuronal gain changes, providing further evidence for the attentional nature of the impact of post-target cues [5]. By increasing the neuronal contrast gain, postcued attention enhances the discriminability between relevant targets and irrelevant noise, resulting in an overall better performance in valid compared to invalid trials. The current experiment, thus, makes clear that the benefits of valid cues found by Sergent et al. (2013) [5] might have indeed been an effect that can only be measured with very low target visibilities.

## 4. General Discussion

In two experiments, we tested and confirmed the view of a perception–memory continuum [18]. According to this view, following target offset, but prior to conscious visual target perception, a malleable representation of the target is open to post-target influences on target perception, such as that of spatial attention [5,6]. In line with this view, we found an influence of attentional guidance through post-target cues on target perception. Such retrospectively triggered modulation of target perception by the guidance of attention to a non-predictive postcue has been reported in a seminal study with targets at threshold [5]. However, so far, it has been open if the same principle can be confirmed with supraliminal targets as implied by the generality of the perception–memory continuum principle. In the current Experiment 1, we, therefore, used clearly visible targets of high contrast. This measure also helps to address another potential complication of the original studies by Sergent et al. (2013) [5] and Thibault et al. (2016) [6]: due to the fact that in the original studies the corresponding modulations were based on targets that were of a weak contrast, it was unclear if, subjectively, targets were not in fact perceived temporally much closer to the post-target cues, but this is unknown as corresponding information on exact target luminance was lacking in Sergent et al. [5].

We also took the opportunity to investigate the generality of the perception–memory continuum principle in another respect. In contrast to prior studies, we did not use a response prompt. Instead, participants were allowed to respond to the targets as fast as they wanted. It is true that, therefore, sometimes responses were given too fast to allow for a post-target cue influence. However, only by leaving out the response prompts could we ensure that the postcueing effects reported in past studies were truly reflective of a general continuity between perception and memory and not merely a consequence of a memory demand imposed by the response prompts. To note, Sergent et al. (2013) [5] used post-target response prompts to ensure cue processing prior to the target judgments in all of the trials, but this implied that their participants were forced to draw on a memory representation of the targets, leaving it open if memory would also be involved in visual perception under more conventional perceptual conditions.

Importantly, with our complementary protocol, we were able to confirm the conclusions of Sergent et al. (2013) [5]. However, in Experiment 1, the decisive experimental evidence differed from that of Sergent et al.: the performance difference was between invalid and neutral (uncued) trials rather than between valid and neutral (double-cueing trials), as in [5]. In Experiment 1, we observed that invalid post-target cues presented away from the target interfered with target processing in comparison to conditions without a cue. This cost by misguided attention cannot be due to direct influences of cue features, such as their lower contrast, on the perception of targets at the cued position [33], as, in the current study, this effect was observed with cues presented away from the targets. In addition, the cost created by the invalid cues in Experiment 1 was likely due to target perception naturally extending to some form of target–memory representation during which post-target cues exerted their influence as participants did not have to wait until a post-target response prompt was presented, and, thus, participants were not required to draw on a memory representation of the target only for task-specific reasons. Instead, the costs of invalid cues created in the present Experiment 1 were most likely due to attention-modulated target perception, as conscious visual perception in general depends on some form of target memory [18]. For example, attentional capture by the invalid cue might have interrupted ongoing spatially specific processing of representations of targets presented away from the cue and, thereby, delayed correct target judgments to some extent [48,49,50]. To note, costs by the invalid post-target cues in the current study were only observed in the RTs, not in ARs. This difference from Sergent et al. (2013) [5] is likely due to the fact that we used supraliminal targets, such that visibility of target orientation and, thus, accuracy itself was not in doubt.

In Experiment 1, we also manipulated target eccentricity to vary task demands. Here, we found that costs by the invalid cues, and, thus, evidence for a modulating impact of attention capture by the post-target cues on target perception, were the same across different eccentricities. This was the case besides decreasing task performance with increasing eccentricity. Thus, one might conclude that the postcueing effect did not indicate contrast gain. However, the nonsignificant effect of target eccentricity on validity could have likewise been due to an insufficient sensitivity of the method as, even with the most eccentric positions, performance was well above the threshold. Therefore, and because the benchmark effect of facilitation in the valid conditions of Sergent et al. (2013) [5] was missing in Experiment 1, we tested the possibility that more evidence of a modulating influence of post-target cues on target perception could be found with a stronger visibility manipulation. This was done in Experiment 2, in which task difficulty was manipulated through noise orientation. In Experiment 2, the postcueing effect was most pronounced for the most difficult conditions (i.e., noise conditions 0° and 15°), a finding in line with contrast gain [37], but only with the short SOA. Thus, the effects of the different difficulty manipulations on postcueing were not the same. This is also interesting in light of the prior observations of Sergent et al. (2013), who studied postcueing only under relatively difficult target-discrimination conditions and, thus, had to leave open the question of if task difficulty was decisive for their found postcueing effect [5]. Based on Experiment 2, this seems to be the case, as our noise-orientation manipulation in that experiment was more similar to the contrast manipulation of Sergent et al. [5] in that stimulus processing at lower eccentricities was not difficult enough per se.

As explained, we attributed the findings in Experiment 2 (of attentional guidance by postcues on the more difficult perceptual conditions) to contrast gain, thereby selectively improving the sensitivity of neurons for low to intermediate stimulus visibility or contrasts [37]. Conversely, in Experiment 2, we did not find such an improvement for highly visible target stimuli embedded in our noise masks, suggesting that overall neuronal responsiveness did not increase at the attended spatial location [34]. Our participants tended to leverage the benefits of postcue-elicited contrast gain to separate a potential target more effectively from noise. This relationship seemed to be nonlinear, which might be the leading reason why the effect was not evident in analyses based on the linear model.

This effect was only visible in the short SOA condition, while there was no evidence for its existence in the long SOA condition. One reason for this might be that participants could utilize the postcue better with the short SOA, as a continuously developing target representation is not yet as advanced (on average) when the cue is presented under long-SOA conditions (see also our discussion on the timing of the long-SOA cues below). This would imply that the long-SOA effect found in past studies [5] must have had another origin. Maybe the delay of the low-contrast representation of the target shifted the target representation indeed closer to the post-cue, even under longer SOA conditions, in past studies. As we manipulated noise mask orientations instead of target contrasts, the same influence was not at work in the current study.

Reduced efficiency of the postcues in long compared to short SOA conditions might have also been due to an attentional blink. The attentional blink describes a reduced ability to report a second target appearing in close temporal proximity to a preceding first target. Participants usually fail to see a second goal-relevant stimulus if it occurs within 200–500 ms from the first target and if a distractor is presented in between successive targets [51,52]. Although the effects are observed with targets presented in rapid succession [53], the attentional blink phenomenon depends on the presence of distractors in between successive targets (cf. lag-1 sparing; [54]). Thus, active rejection of distractors, here of the irrelevant and non-predictive postcues, is responsible for the attentional blink phenomenon [55] and would lead to less pronounced postcueing effects with more effectively rejected cues under longer than under shorter target–cue interval conditions (due to a more likely switch between boosting of the targets versus bouncing the distracting cues) in the case of the long SOA than the short SOA of the present study.

Another reason for increased contrast gain in short SOA conditions only might be the maintenance of the cued information in a more active state in visual working memory (VWM) in the short SOA condition [20]. This might then retrospectively enhance the accessibility of the correct target representation, leading to differences between short compared to long SOA conditions. Although this explanation is not one in terms of attention capture, it relies on spatial selectivity impacting the continuum of perceptual and memory processes, too [50,56,57,58]. To summarize, by manipulating target visibility across a broad range of performance levels in Experiment 2, we were able to demonstrate the beneficial effect of valid postcues on participants’ target perception. Crucially, we have shown that this process operates based on contrast gain rather than response gain and is more visible under short SOA conditions. This might be due to diverse mechanisms, such as better cue utilization due to attentional phenomena like the Attentional Blink or improved integration of all stimuli in VWM with shorter than longer stimulus–stimulus intervals. Further research should focus on the origins of the SOA’s modulating effect on postcueing, while simultaneously confirming the influence of contrast gain.

Theoretically, it could be objected that, by using supraliminal stimuli, the current Experiment 1 would have prevented any impact of the post-target cue on target perception and that, hence, any influence the post-target cue in the current study may have had must have been of a non-perceptual origin. However, this argument draws a relatively artificial line between qualitative changes of the conscious percept on the one hand, and between quantitative costs (or savings) in target-processing times on the other hand. According to this distinction, “reviving” the access to the perception of an otherwise lost liminal stimulus by a valid post-target cue would count as a qualitative change of perception. In contrast, a reaction-time cost incurred by an invalid cue would reflect a mere quantitative processing difference. To understand the artificiality of this distinction, however, consider two findings from the literature: confidence effects in visibility studies [59,60] and perceptual latency priming through peripheral cues [61,62]. Starting with the former, reports about visual targets jointly reflect independent influences of target visibility and confidence [59]. This means that lack of confidence can contribute to the failure to report an otherwise seen liminal target and that a post-cue could have its impact on reports of liminal targets via confidence rather than target visibility. Thus, it is naïve to assume that the impact of the post-cue on the visibility of liminal stimuli in studies such as that of Sergent et al. (2013) [5] must have reflected a qualitative change of perception. It might in fact rather be an impact on participants’ confidence in the perception of an otherwise unaltered perceptual object [59].

Now turning to the RT cueing effect, peripheral cues preceding the target are known to affect the quality of conscious target perception. They affect the point in time at which the target is consciously seen, i.e., a valid cue speeds up target perception and an invalid cue delays target perception, as reflected in perceptual order judgments [61]. Thus, it is equally naïve to assume that the temporal delay in target reports caused by invalid post-cues must be of a non-perceptual origin. Together, these considerations make clear that neither of these approaches to the study of the perception–memory continuum is air-tight, but the upshot should be that the corresponding research should be carried out with these reservations in mind, rather than postponed until an indefinite future point in time at which we know how to approach these fundamental problems of psychophysics.

## 5. Conclusions

In the current study, we tested and confirmed the view of a continuum between perceptual and memory representations in the visual domain [18]. Specifically, we demonstrated the generality and robustness of the impact of attentional capture by post-target cues on the perception of cue-preceding visual targets. Although our results partly differed from previous research that found a benefit of valid compared to invalid postcues, in our Experiment 1, we demonstrated a spatial postcueing effect by interference through attentional capture by cues drawing attention away from the target compared to uncued trials. The results, thus, show that visual target perception is not “encapsulated” and shielded against distraction following the target, but is vulnerable to visuo-spatial attentional effects elicited by visual events occurring after the target has been presented. More specifically, our results show that corresponding influences found by Sergent et al. (2013) [5] are not restricted to the perception of liminal stimuli and that they generalize to supraliminal stimuli. We have additionally shown that postcueing effects occur at least partly by contrast gain rather than response gain and can be found in both speed and accuracy domains. Future research could additionally investigate whether these findings transfer to other modalities and whether cross-modal postcueing is possible; it would also be beneficial to explore whether these principles can be demonstrated in a more applied context, bridging the gap between basic research and tangible, everyday applications.

## Data Availability

The data presented in this study are openly available on the Open Science Framework at https://doi.org/10.17605/OSF.IO/825ED (accessed on 17 December 2023).

## Appendix A

**Table A1:** Experiment 2: Three-way ANOVA performed with *d*’ as dependent variable. Results with a *p* value < 0.05 are marked with *. *p* values for the factor Orientation were Greenhouse–Geisser-adjusted.

Effect	*F* (df)	*p*	$\eta_{p}^{2}$	BF_Incl_
(Intercept)	269.32 (1, 23)	<0.001 *	0.92	
Validity	2.24 (1, 23)	0.148	0.09	0.323
SOA	0.45 (1, 23)	0.508	0.02	0.176
Orientation	47.68 (4, 92)	<0.001 *	0.67	>100
Validity × SOA	0.09 (1, 23)	0.762	<0.01	0.18
SOA × Orientation	2.48 (4, 92)	0.050	0.10	0.404
Validity × Orientation	1.60 (4, 92)	0.180	0.07	0.175
Validity × SOA × Orientation	0.89 (4, 92)	0.472	0.04	0.216

**Table A2:** Experiment 2: Comparison of logistic and Gaussian models fitted for valid and invalid conditions, and for short (150 ms) and long (400 ms) conditions, using Akaike Information Criterion (AIC), as well as Bayesian Information Criterion (BIC).

SOA	Validity	AIC_Log_	BIC_Log_	AIC_Gauss_	BIC_Gauss_
150 ms	valid	135.0752	139.7874	135.0790	139.7910
	invalid	137.3606	142.0728	137.3626	142.0748
400 ms	valid	136.4380	141.1502	136.4400	141.1522
	invalid	137.8132	142.5254	137.8144	142.5266

Note. SOA = Stimulus onset asynchrony.

**Table A3:** Experiment 2: psychometric parameter estimations fitted to individual participants’ mean accuracy rate with varying stimulus orientation (from 0° to 90°), one model for each validity condition (valid and invalid condition), and stimulus–onset asynchrony (SOA; 150 ms and 400 ms).

SOA	Validity	α	β	γ	λ	LL
150 ms	valid	−12.2038	0.1109	<0.001	0.1878	−63.5376
	invalid	12.2555	0.2162	0.6175	0.19	−64.0803
400 ms	valid	−8.7449	0.1633	<0.001	0.2	−64.2190
	invalid	−9.1801	0.1407	<0.001	0.2	−64.9066

**Table A4:** Experiment 2: Psychometric parameter estimations fitted to individual participants’ mean accuracy rate with varying stimulus orientation (from 0° to 90°), one model for each validity condition (valid, uncued, and invalid conditions), aggregated over stimulus–onset asynchronies.

Validity	α	β	γ	λ	LL
Valid	−11.3916	0.1229	<0.001	0.1933	−63.8869
Invalid	−8.3230	0.1357	<0.001	0.1968	−64.8702
uncued	6.9628	0.1689	05742	0.1950	−64.8172
